# Evaluation of Micronutrients and Pro-Inflammatory Cytokines Levels in Nutritionally Deprived Children—A Tertiary Care Hospital-Based Study

**DOI:** 10.3390/nu15234865

**Published:** 2023-11-22

**Authors:** Malvika Mishra, Alok Raghav, Prashant Tripathi, Yashwant Kumar Rao, Desh Deepak Singh

**Affiliations:** 1 Amity Institute of Biotechnology, Amity University Rajasthan, Jaipur 303002, India; malvikamishra2121@gmail.com; 2 Department of Anatomy and Cell Biology, Lee Gil Ya Cancer and Diabetes Institute, Gachon University, Incheon 21999, Republic of Korea; alokalig@gmail.com; 3 Department of Biochemistry, Ganesh Shankar Vidyarthi Memorial Medical College, Kanpur 208002, India; prashantamu@gmail.com; 4 Department of Pediatrics, Ganesh Shankar Vidyarthi Memorial Medical College, Kanpur 208002, India; ykraoneo@yahoo.co.in

**Keywords:** severe acute malnutrition, antibiotics, inflammatory cytokines, ICP, CRP, anthropometric parameters

## Abstract

Background: Severe acute malnutrition (SAM) is a significant public health problem in developing countries, including India, where a significant proportion of children suffer from malnutrition. Objective: This research aims to investigate the factors contributing to severe acute malnutrition (SAM). Additionally, the study seeks to explore the relationship between micronutrient levels and pro-inflammatory cytokines in SAM children with and without clinical complications. Furthermore, the effectiveness of antibiotic treatment in SAM children without complications is evaluated. Methods: The study involved three groups comprising 66 subjects each: a healthy control group, SAM children with complications, and SAM children without complications. Blood samples were collected, and various analyses were conducted, including biochemical, hematological, micronutrient, and pro-inflammatory marker quantification. The data were analyzed using SPSS version 22.0. Results: The results indicate that the levels of IL-6, CRP, and TNF-α were significantly higher in the SAM group with complications compared to both the control group and the SAM group without complications. Zinc and copper levels were significantly lower in both SAM groups compared to the control group, and a negative correlation was observed between zinc levels and inflammatory markers. The study also assessed the efficacy of antibiotic treatment in SAM children without complications by comparing their weight, height, weight-for-height, and weight-for-age at baseline and after a 15-day follow-up period. Significant improvements in these parameters were observed in both the group receiving antibiotic treatment and the group not receiving antibiotic treatment. Conclusion: The findings suggest that a combination of antibiotic treatment and nutritional support can lead to significant clinical improvements in SAM children without complications. This study has important implications for the management and treatment of SAM in India and other developing countries.

## 1. Introduction

India holds the dubious distinction of being the birthplace of a third of the world’s entire population of malnourished children. Around 32.1% of India’s children are underweight, 35.5% are stunted, and 19.3% are wasted, as per the National Family Health Survey-5 (NFHS-5) report [1]. The current classification of malnutrition is based on body size or the presence of edema, which does not provide information about the specific nutritional deficiencies that an individual may have. SAM, as defined by WHO-UNICEF (World Health Organization—United Nations International Children’s Emergency Fund), includes severe wasting that is defined by anthropometric criteria as a weight-for-height Z-score below −3, a mid-upper arm circumference (MUAC) < 11.5 cm (115 mm), or bilateral pitting edema in children aged 6 to 59 months of age (WHO, 2013). According to WHO (World Health Organization) recommendations, complicated SAM children are unable to drink or breastfeed, fail the ready-to-use therapeutic food (RUTF) appetite test, and exhibit clinical features such as septic shock, hypoglycemia, hypothermia, skin infection, respiratory or urinary tract infection, bipedal edema, severe wasting, dehydration, diarrhea, tuberculosis, etc., while children with uncomplicated SAM are clinically well, alert, and have retained their appetite and can be managed as outpatients with a course of oral antibiotics. According to the 2006 report given by UNICEF, poor breastfeeding practices, an insufficient diet, frequent infections, and the delayed introduction of complementary foods into the diet were some of the leading causes of childhood malnutrition [2]. Micronutrient deficiencies, including vitamins, minerals, and trace elements, are prevalent among children with SAM. Understanding the specific micronutrient profiles in SAM children, particularly about treatment response, is essential for tailoring nutritional interventions and optimizing treatment protocols. Zinc (Zn) and copper (Cu) are essential micronutrients that play crucial roles in the pathophysiology and management of severe acute malnutrition (SAM) in children. These are involved in various physiological processes, including growth, immune function, antioxidant defence, and energy metabolism. In the context of SAM, disruptions in Zn and Cu status can have profound implications for disease severity, treatment response, and long-term outcomes. Several studies have highlighted the importance of zinc supplementation in the management of SAM. Zinc deficiency in children is associated with growth retardation, increased susceptibility to infection, and decreased immunocompetence, therefore contributing to a large amount of childhood morbidity and premature mortality [3]. Zinc supplementation has been shown to improve weight gain, reduce the duration of diarrhea, enhance immune function, and reduce the risk of mortality in children with SAM [4]. Furthermore, zinc supplementation has been found to enhance the efficacy of therapeutic feeding protocols, leading to more rapid recovery and improved nutritional status [5]. Similarly, copper deficiency can also occur in children with SAM, although it is less extensively studied compared to zinc deficiency. Copper plays a crucial role in collagen synthesis, iron metabolism, and antioxidant defence mechanisms. In addition to micronutrients, pro-inflammatory cytokines also play a pivotal role in the pathophysiology of SAM. Inflammatory processes can disrupt normal growth and metabolism, exacerbate wasting, and contribute to the development of clinical complications. Elevated levels of proinflammatory cytokines have been observed in children with SAM and are associated with disease severity. Increased levels of IL-6 and TNF-α have been specifically reported in SAM children compared to healthy controls [6,7]. These cytokines are involved in the systemic inflammatory response, leading to the breakdown of muscle protein, increased resting energy expenditure, and reduced appetite, ultimately contributing to the wasting seen in SAM [8]. In SAM children, zinc deficiency has been linked to elevated levels of pro-inflammatory cytokines such as interleukin-6 (IL-6) and tumor necrosis factor-alpha (TNF-α) [9,10]. Studies have reported that zinc supplementation in SAM children is associated with decreased levels of pro-inflammatory cytokines, suggesting a potential regulatory role for zinc in modulating the inflammatory response [11,12]. Similarly, copper deficiency may contribute to increased proinflammatory cytokine production and oxidative stress, leading to a dysregulated immune response [13]. Although direct evidence in SAM children is scarce, studies in other populations have demonstrated that copper supplementation can modulate the production of pro-inflammatory cytokines such as TNF-α and IL-6 [14,15].

The objective of this study was to identify factors contributing to SAM, investigate the correlation between micronutrient levels and pro-inflammatory cytokines in SAM children with and without clinical complications, and evaluate the effectiveness of antibiotic treatment in SAM children without clinical complications. Overall, this study represents a crucial step towards a deeper understanding of the intricate mechanisms underlying severe acute malnutrition. By bridging the knowledge gaps surrounding micronutrients, pro-inflammatory cytokines, and treatment optimization, we strive to contribute to the global efforts aimed at combating SAM and alleviating its devastating impact on the world’s most vulnerable populations.

## 2. Materials and Methods

This cross-sectional study was conducted at the Department of Pediatrics and Biochemistry of Ganesh Shankar Vidyarthi Memorial Medical College, Kanpur (GSVM) and the Department of Biotechnology, Amity University, Rajasthan, Jaipur. The study aimed to investigate the contributing factors for SAM, the relationship between micronutrient levels and pro-inflammatory cytokines, and clinical complications in children with severe acute malnutrition (SAM).

### 2.1. Sample Size

The eligible study participants were calculated to be 60 in each group, at a power of 95% and a level of significance of 5%. Taking 10% non-response, the sample size came out to be 66. The study group comprised three subgroups: 66 healthy control subjects, 66 SAM children with clinical complications, and 66 SAM children without clinical complications. Study subjects were selected based on specific inclusion and exclusion criteria among those who presented at the outpatient department (OPD) or inpatient department (IPD) of the Department of Pediatrics, GSVM Medical College, Kanpur, during the first visit.

### 2.2. Inclusion Criteria for Subject Recruitment

According to WHO criteria for SAM, children aged 6 to 59 months with uncomplicated SAM were approached through house-to-house visits in an area with Anganwadi workers, whereas children with complicated SAM were recruited from the OPD and IPD of the hospital for maximum inclusion of eligible children with the following inclusion criteria:

1. Weight for height below 3 standard deviations (SD or Z scores)
2. Malnutrition with bilateral pedal edema or visible severe wasting
3. Mid-upper arm circumference (MUAC) < 115 mm

### 2.3. Exclusion Criteria

**Figure 1:**
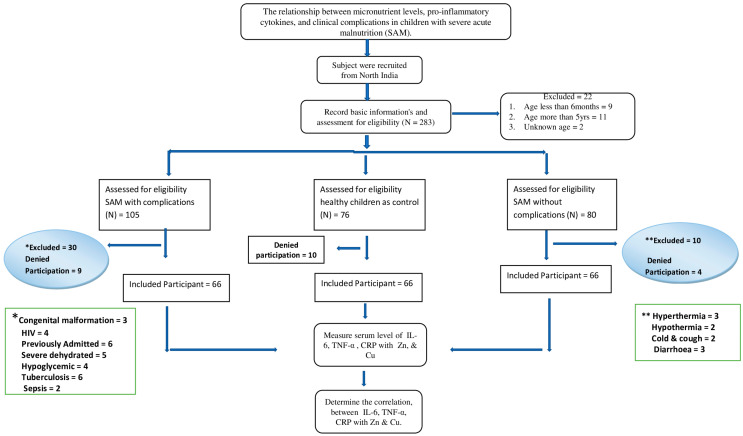
CONSORT diagram outlining how participants were recruited. * and ** are different exclusion criteria for both groups.

i. Children aged <6 months and >5 years, because infants under 6 months rely solely on breast milk or formula for their nutritional needs. The introduction of complementary foods typically begins around 6 months of age. The majority of the research that was available at the time of the WHO guidelines was geared toward children 6 months and older, or
ii. Congenital malformation, which was assessed through a physical examination conducted by pediatricians; chronic diseases other than HIV and TB, confirmed through the identification of signs and symptoms; and children who underwent an assessment to determine the presence of palmar pallor. Those who exhibited extremely pale palms, appearing white, were identified as having severe anemia and were subsequently excluded from the study or analysis.
iii. For children with uncomplicated SAM, a history was taken to assess for symptoms suggesting that the child was not clinically well (cough, shortness of breath, diarrhea, fever, and anorexia); children assessed as being clinically unwell based on symptoms and any recent use of antibiotics within the past 14 days, determined through interviews with parents and cross-referencing with previous medical records if the baby had been admitted during that period, were excluded from the group of uncomplicated SAM. (Figure 1).

### 2.4. Baseline Information

Anthropometric measurements were obtained from the study participants using a standardized protocol recommended by WHO/UNICEF (2009) to ensure accuracy and consistency. Demographic data and risk factors related to SAM were collected using a pre-tested questionnaire to obtain comprehensive information from the study participants. The questionnaire was designed to capture data on factors such as age, sex, socioeconomic status, dietary habits, and health status, which are known to influence the development and progression of malnutrition. To ensure data quality, the questionnaire was pretested on a small sample of 10 children to identify and address any issues with the questionnaire design and administration.

### 2.5. Sample Collection and Processing

In the study, 5 mL of venous blood samples and 3 mL of blood were collected from anemic study subjects before any treatment or management intervention. Blood was collected in two different vials: one with EDTA and one without (a plain vial). Serum was then separated through centrifugation, and liver function tests (LFT), kidney function tests (KFT), and hematological analysis (CBC) with whole blood collected in an EDTA vial using a cell counter (Medonic, Model M-20 cell counter, Boule Medical AB, Spanga, Sweden) were performed using kit (Q-line biotech) methods. The serum concentrations of zinc and copper were determined using ICP spectrometry (Avio 560 Max ICP Optical Emission Spectrometer, Perkin Elmer, Waltham, MA, USA). In addition, the serum concentration of cytokines IL6 and TNF-α was determined using immune-enzymatic assays using ELISA kits (VECTOR-BEST, Russia, Catalog no. 8768 and 8756) and CRP (Xema, Moscow, Russia), respectively.

### 2.6. Quantification of Micronutrients Using Inductively Coupled Plasma Mass Spectrometry

Venous blood samples (2 mL) were obtained from the participants, and serum was extracted within 3 h (3500 rpm, 35 min). Samples with hemolysis, lipids, or jaundice were not analyzed. To prepare the mixed solution for analysis, one milliliter of 5% HNO_3_ solution was added to one milliliter of blood sample, followed by centrifugation at 12,000 rpm for 5 min to extract the supernatant. Next, 3.5 mL of 1% HNO_3_ solution was added to 0.5 mL of the supernatant, and the mixture was centrifuged for 2 min at 2500 rpm. The resulting samples were then kept at room temperature for 1 min before being subjected to ICP-MS analysis (ICPMS-2030, Shimadzu, Japan).

### 2.7. Quantification of Serum IL-6, TNF-α, and CRP

The levels of IL-6, TNF-α, and CRP in the blood were measured using commercially available enzyme-linked immunosorbent assay (ELISA) kits. The ELISA test involved preparing reagents, standards, and serum samples according to the manufacturer’s instructions for the relevant ELISA kit (VECTOR-BEST, Novosibirsk, Russia, Catalog no. 8768). The microliter wells on each ELISA kit plate were coated with specific antibodies that recognized an antigenic site on a particular cytokine molecule. The measured values of IL-6 and CRP were expressed in units of pg/mL and mg/L, respectively. The sensitivity of the assay was less than 2 pg/mL for both IL-6 and CRP, indicating that it was capable of detecting low levels of these cytokines. There was no cross-reactivity with other cytokines present in the serum samples.

### 2.8. Treatment Optimization

Children 6–59 months of age with uncomplicated SAM presenting to nutritional programs at eligible health centers in Kanpur District were randomized to a single dose of an oral 7-day course of amoxicillin at baseline. Apart from the administration of antibiotics, all children received standard outpatient treatment for uncomplicated SAM as specified in the guidelines of the government of India. The children with uncomplicated SAM were randomly divided into two groups: 

The management of SAM in both groups, apart from the administration of antibiotics, was the same in terms of diet supplements and other interventions, as subjects were divided into the two groups, so we did not collect data on RUTF and diet.

Group I was the control group, receiving amoxicillin for a week at a dose of 40 mg/kg/day.

Group II was the intervention group that willingly did not take antibiotics.

Enrolled children were followed on the 15th day of follow-up visits from the day of enrollment. Anthropometric parameters were collected at the baseline and 15th day follow-up visits, and weight gain and nutritional recovery over a 2-week period were compared by another group. To monitor adherence to the treatment protocol, the administration of antibiotics was carefully monitored by healthcare providers (Anganwadi) as their major task, as per government guidelines. The children in Group I were supervised to ensure they received the prescribed dose of amoxicillin for the specified duration. The inclusion of Group II in our study was based on their preference not to take antibiotics at the initial stage. Despite their reluctance, we included them to assess the effectiveness of the standard outpatient treatment without antibiotics for uncomplicated severe acute malnutrition (SAM). This allowed us to compare the outcomes between Group I (the control group receiving amoxicillin) and Group II (the intervention group not taking antibiotics) and evaluate the potential of non-antibiotic treatment as an alternative approach.

### 2.9. Statistical Analysis

The statistical analysis for this study was conducted using the Statistical Package for Social Sciences (SPSS) software, version 22.0, which is a commonly used statistical tool for analyzing data in the social and health sciences. Descriptive statistics such as mean and standard deviation (SD) were used for continuous variables, while percentages were used for categorical variables. To determine the differences between categorical variables, the chi-square test was employed, and for continuous variables, the analysis of variance (ANOVA) was used. A *p*-value of less than 0.05 was considered statistically significant.

## 3. Results

The results of the study presented a comprehensive analysis of multiple variables related to the enrolled patients, as shown in Figure 1.

In terms of demographic and socioeconomic characteristics, the gender distribution revealed variations across the groups, with the control group comprising more females and the complicated SAM group having a higher proportion of males. The majority of participants in each group belonged to low-income groups (74.2% of children with uncomplicated SAM and 53% of children with complicated SAM), with none falling into the high-income category. Additionally, most participants were from nuclear families in each of the three groups (Table 1). 

When considering maternal characteristics, the age at birth showed a predominant trend of mothers falling between the 20–30 (77.2%, 81.8%, and 89.3%) age range across all three groups. First-born children were prevalent, and mothers generally had gravidity and parity of less than 5. Most mothers reported taking special meals during pregnancy, and a significant proportion of them were aware of their child’s malnutrition status. The majority received antenatal care and supplementation with iron and folic acid tablets (Table 2).

The analysis of factors associated with severe acute malnutrition (SAM) revealed several noteworthy findings. Child age emerged as a significant factor, with the 6–20-month age group showing a higher proportion of complicated SAM cases (74.2%). Initiation of breastfeeding also played a role, as delayed breastfeeding was about 77.2% in children with complicated SAM. Feeding practices such as bottle feeding, pre-lacteal feed, and the number of daily meals showed significant associations with SAM, with higher proportions observed in the complicated SAM group. Water consumption and urination frequency during the daytime were also linked to SAM status (Table 3).

**Figure 2:**
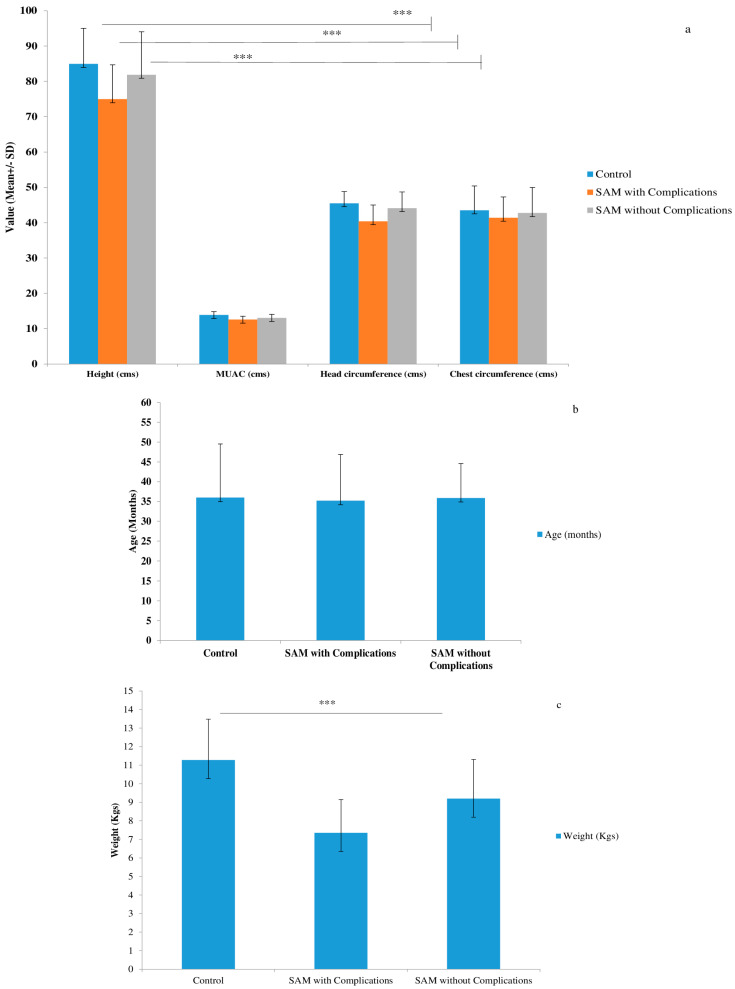

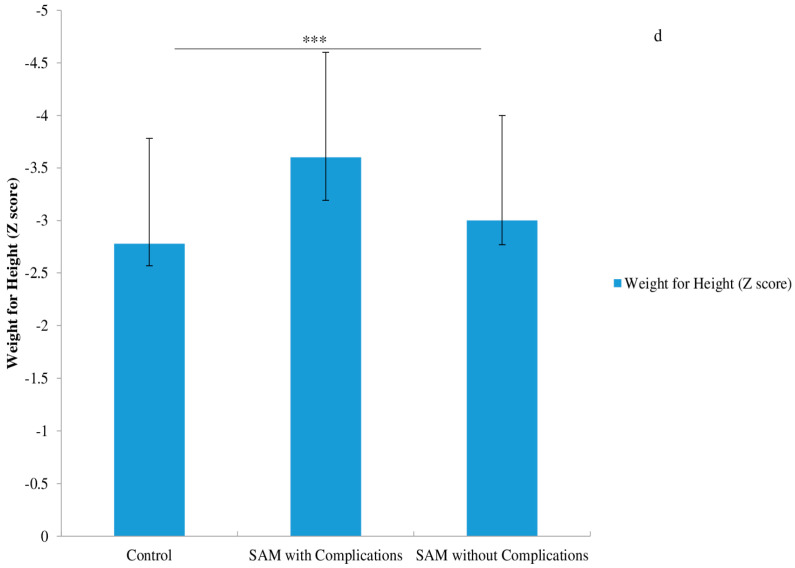
Anthropometric characteristics: (**a**) showing the comparison within study groups of height, chest circumference, and head circumference; (**b**) age (months); (**c**) weight (Kgs); and (**d**) weight for height (Z score) (mean + SD) using an ANOVA. *** Standard deviation from mean values.

Anthropometric characteristics demonstrated the impact of severe acute malnutrition on growth and development. Weight-for-height Z scores differed significantly among the three groups, indicating compromised growth in SAM cases. Other anthropometric parameters, including MUAC, head circumference, and chest circumference, also showed significant differences (Figure 2).

Blood and organ function analyses shed light on the physiological effects of severe acute malnutrition. In terms of complete blood count, Hb levels, platelet count, total RBCs, and MPV were significantly lower in the children with complicated SAM (7.75 + 1.02, 3.36 + 0.96, 4.07 + 0.69, and 8.06 + 0.96), respectively. Kidney function tests revealed higher serum urea and creatinine levels in the SAM groups compared to the control group. Liver function tests exhibited elevated levels of various parameters in the SAM groups, including bilirubin, protein, albumin, SGOT, and SGPT (Table 4). 

**Figure 3:**
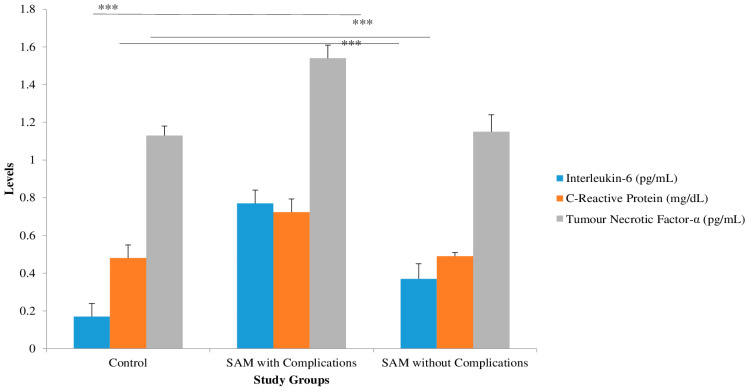
Pro-inflammatory cytokines levels in severe acute malnutrition children with and without complications (mean + SD) using an ANOVA. *** Standard deviation from mean values.

Inflammatory markers IL-6 and CRP, as well as TNF-α (0.77 + 0.07, 0.72 + 0.07, and 1.54 + 0.00, respectively), were significantly higher in the children with complicated SAM (Figure 3). 

**Figure 4:**
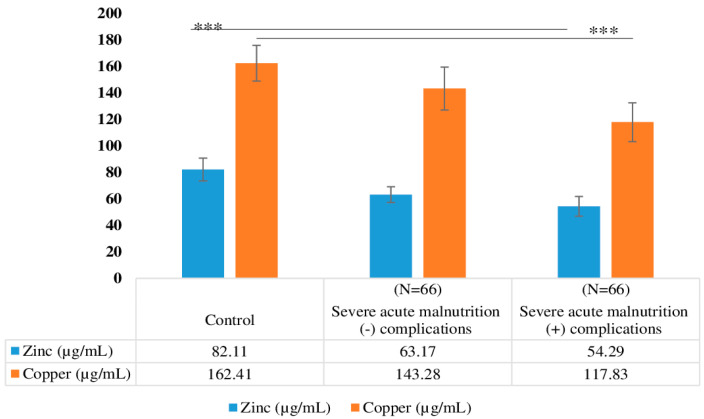
Micronutrient level among the SAM children with (*n* = 66) and without complications (*n* = 66) (mean + SD) using an ANOVA. *** Standard deviation from mean values.

Micronutrient levels, such as zinc and copper, also varied significantly among the groups (Figure 4).

The baseline weight was 7.31 ± 1.60 kg, which increased significantly to 7.90 ± 2.00 kg at the 15th day after antibiotic supplementation for participants who received antibiotic treatment. The weight, height, weight-for-height Z score, and weight-for-age Z score for participants who did not receive antibiotics were 7.60 ± 1.77 kg, 74.73 ± 9.80 cm, −2.29 ± 1.70, and −4.00 ± 2.40. (Table 5). 

These findings collectively provide valuable insights into the various factors associated with severe acute malnutrition in children. Child age, initiation of breastfeeding, feeding practices, water consumption, and blood and organ function parameters all contribute to the development and severity of SAM. Understanding these factors is crucial for implementing targeted interventions to improve child health outcomes and combat malnutrition.

## 4. Discussion

The findings revealed that female gender, low household income, young maternal age, and poor hygiene practices were some of the contributing factors that favored the development of SAM.

Our results are in complete agreement with the results of the previously published study by Ghimire U et al., in which they observed that the percentage of females was among the highest in severely acutely malnourished children (5.8%) [16]. Further, it was also found that the female gender was associated with stronger risk factors for SAM [17]. The outcomes of our investigation align with previous research conducted by Mukuku et al. (2019) [18], which highlighted low birth weight, a history of diarrhea, insufficient daily meals, early discontinuation of breastfeeding, early introduction of complementary diets, maternal age below 25 years, parity less than five, and more than two children under the age of five as risk factors for severe acute malnutrition (SAM). The results of our study showed that child age, childbirth history, and birth weight contribute significantly to severe acute malnutrition. The results of the previously published study were also aligned with the observations made in our study. The authors also showed that average perceived birth weight [(AOR = 0.048, 95% CI: 0.015, −0.148)] and large perceived birth weight [(AOR = 0.023, 95% CI: (0.002, −0.271)] were significant factors associated with severe acute malnutrition [19].

The study conducted by Ghimire et al. (2020) [16] highlights the significant role of household size, household food access, and the child’s age as crucial predictors of severe acute malnutrition. The World Health Organization (WHO) recommends exclusive breastfeeding for the first six months of life as the gold standard for ensuring optimal child health. However, findings from the Hungama survey indicate that only 40% of Indian infants received exclusive breastfeeding during the first six months of life, highlighting a potential risk factor for severe acute malnutrition (SAM). This result is consistent with the findings reported by Hong et al. (2006) [20] in their research, which also showed low rates of exclusive breastfeeding in India. The implications of these findings are significant, as exclusive breastfeeding plays a crucial role in providing essential nutrients to infants and protecting them against infections, which are known risk factors for SAM. The results of the present study also concluded that there is a strong correlation between weight and head circumference, triceps skinfold for age (z score), and subscapular skinfold for age (z score), along with BMI, among SAM children with and without complications. The results of the present study showed that the body composition of SAM children with complications is significantly associated with anthropometric measurements. The results are in agreement with the previous study that showed WHZ, WAZ, and BMI were significantly associated with fat free mass index, while MUACZ was significantly associated with both fat mass index and fat free mass index. Children with both severe wasting and severe stunting had significantly lower FFMI compared to those who were only severely wasted [21]. A comparison of biochemical parameters, including complete blood count, kidney function test, and liver function test, between all groups shows there were significant differences in the study. Similarly, in another study, the authors compared the clinical characteristics, biochemical features, and health and nutrition histories of nonedematous children with SAM who had (1) low WHZ only, (2) both low WHZ and low MUAC, or (3) low MUAC only, and found that serum and clinical parameters were found to be significant, including signs of severe wasting, dehydration, serum ferritin levels, and caretaker-reported health deterioration, and were replicated across study sites [22].

Despite the high mortality burden, predominantly due to infections, the underlying pathogenic pathways remain poorly understood. Intestinal and systemic inflammation are heightened in children with SAM.

Patients with severe acute malnutrition (SAM) are known to be susceptible to secondary immune deficiency and often exhibit elevated systemic inflammation markers that are correlated with diarrhea and mortality [23]. In our study, we observed a significant increase in the levels of interleukin-6 (IL-6), tumor necrosis factor-alpha (TNF-α), and C-reactive protein (CRP) among children with complicated severe acute malnutrition (SAM). Our results are in complete agreement with previously published study observations that showed IL-6 values were increased in patients at nutritional risk (34.9%) compared with those who were well-nourished [7.12 (0.58–34.23) pg/mL vs 1.63 (0.53–3.43) pg/mL; *p* = 0.02], correlating inversely with triceps skinfold-for-age z-score (rs = −0.61; *p* < 0.001) [24]. Micronutrient deficiencies, particularly vitamins A, D, E, and B12, folic acid, zinc, and iron, have been well-established risk factors for the development and progression of SAM. These deficiencies can impair immune function, retard growth, and adversely affect cognitive development, among other adverse outcomes [25]. Children suffering from severe and moderate malnutrition may have inadequate levels of copper (Cu), zinc (Zn), and iron (Fe), which are essential for their nutritional recovery. In children who were recovering from malnutrition, there was a deficiency of copper, which is a vital metal required for various enzymes necessary for life [26]. Ahsan AK (2021) and their co-workers conducted a study that suggested a four-fold higher prevalence of zinc deficiency in cases of malnutrition compared to the control group [27]. Mayo-Wilson et al. demonstrated a notable positive impact of zinc supplementation on both height and weight in children ranging from 6 months to 12 years of age. [28]. In our study, we observed a significant negative correlation between zinc and TNF-α in both SAM children with complications and those without complications. Additionally, copper showed a significant positive association with TNF-α in both groups of SAM children, with a significant positive association with IL-6 observed in SAM children without complications. Awatif (2017) [29] reported that zinc shows a negative correlation with inflammatory markers. Lindenmayer reported that zinc deficiency could worsen systemic inflammation, which is known to contribute to stunting [30].

In the management of SAM, nutritional rehabilitation often involves the use of antibiotics to address underlying infections and improve clinical outcomes. However, the efficacy of antibiotics in treating SAM children without clinical complications remains a subject of debate [31]. In our study, we found that antibiotic treatment in SAM children without clinical complications resulted in significant improvements in weight, weight-for-height Z score, and weight-for-age Z score. After 15 days of follow-up, there was a significant increase in the children’s weight from baseline. Furthermore, without antibiotic treatment, there were significant improvements in the weight-for-height Z score and the weight-for-age Z score. These findings were supported by statistically significant differences in the baseline and follow-up values for weight, weight-for-height Z score, and weight-for-age Z score. However, in 2016, a randomized trial was conducted by Médecins Sans Frontières (MSF) in Niger [32] to assess the effect of routine amoxicillin use, as compared with placebo, on nutritional recovery in children with uncomplicated SAM. A total of 1199 in the amoxicillin group and 1200 in the placebo group were studied. Baseline characteristics were similar; one confirmed case of HIV was included. The SAM recovery rate was 64%. The prevalence of infection among uncomplicated cases in this study (*n* = 1000 sub-groups) was low. No significant benefit of routine amoxicillin use was found with respect to nutritional recovery. In terms of secondary outcomes, amoxicillin use was associated with a significantly shorter time to recovery (28 vs. 30 days); a reduced risk of death among children 24 months of age; a decreased risk of inpatient transfer for complications and acute gastroenteritis; and accelerated gains in weight and MUAC. These findings inform the evidence for context-specific recommendations regarding routine antibiotic use in uncomplicated SAM.

It is rare to see a study including all three groups, and this allows for differences in micronutrients and biomarkers due to clinical complications to be disaggregated from those due to SAM alone and for both to be characterized relative to appropriate control children growing up in the same communities. SAM is widely recognized as a life-threatening condition that contributes to unacceptably high child mortality in low- and middle-income countries; studies such as this are much needed to improve our understanding of SAM etiology. Further, this study spans anthropometric, socioeconomic, feeding practice/maternal care behavior, and laboratory outcome measures that have previously been shown to be associated with clinical outcomes for children with SAM but are not always possible to evaluate simultaneously. The study was conducted in a specific setting: the same rural block and a tertiary care hospital. The findings may not be directly applicable to children with severe acute malnutrition in other healthcare settings or different geographical regions. Since this study had a short follow-up and was conducted on a small number of patients, further studies, including multicentric trials with longer follow-up, are needed for standardization of the protocols and management of SAM children.

## 5. Conclusions

Overall, the study highlights the importance of proper sanitation and hygiene practices, family household income, child age, and the initiation of breast feeding, bottle feeding, pre-lactal feeding, and the number of daily meals among the major contributing factors favoring the SAM pathogenesis. Also, quantification of micronutrients and pro-inflammatory factors can be part of the effective management of SAM to improve children’s nutritional status and prevent severe complications. It is imperative to conduct additional studies, such as multicenter trials with prolonged follow-up, to standardize the protocols concerning the use of antibiotics in children with uncomplicated SAM. 

## Figures and Tables

**Table 1:** Socio-demographic characteristics of study participants.

Variables	Characters	Control (*n* = 66)	Uncomplicated SAM (*n* = 66)	Complicated SAM (*n* = 66)	χ^2^/*p*-Value
Sex	Male	27 (41%)	25(37.8%)	34(51.5%)	2.75/0.25
	Female	39 (59%)	41(62.1%)	32(48.4%)	
Socioeconomic status	High-Income Group (HIG)	0	0	0	-
	Middle Income Group (MIG)	4 (6.0%)	3 (4.5%)	3(4.5%)	
	Low Income Group (LIG)	46 (69.6%)	49 (74.2%)	35 (53.0%)	
	Slum	16 (24.2%)	14 (21.2%)	28 (42.4%)	
Family type	Nuclear	36 (54.5%)	35 (53.0%)	41(62.1%)	1.27/0.52
	Joint	30 (45.4%)	31 (46.9%)	25 (37.8%)	
Household income (monthly)	<5000	37 (56.0%)	33 (50%)	17 (25.7%)	13.77/0.00
	>5000	29 (43.9%)	33 (50%)	49 (74.2%)	
Toilet facility	Open field	3 (4.5%)	4 (6.0%)	2 (3.0%)	1.26/0.86
	Community	14 (21.2%)	15 (22.7%)	12 (18.1%)	
	Private toilet	49 (74.2%)	47 (71.2%)	52 (78.7%)	
Economic background	Above Poverty Line (APL)	30 (45.4%)	41 (62.1%)	15 (22.7%)	1.29/0.59
	Below Poverty Line (BPL)	36 (54.5%)	25 (37.8%)	51 (77.2%)	
Source of drinking water	Draw well	0	0	0	-
	Tube	0	0	0	
	Community tap	0	11(16.6%)	11 (16.6%)	
	Individual tap	19 (28.7%)	14 (21.2%)	13 (19.6%)	
	Filter/packed water	0	0	0	
	Reverse Osmosis plant	6 (9.0%)	1 (1.5%)	2 (3.0%)	
	Hand pump	41 (62.1%)	38 (57.5%)	39 (59.0%)	
	Other	0	2 (3.0%)	1 (1.5%)	

**Table 2:** Mother History of study participants.

Variables	Characters	Control (*n* = 66)	Uncomplicated SAM (*n* = 66)	Complicated SAM (*n* = 66)	χ^2^/*p*-Value
Mother age at birth (years)	<19	5 (7.5%)	3 (4.5%)	2 (3.0%)	3.74/0.44
	20–30	51 (77.2%)	54 (81.8%)	59 (89.3%)	
	>30	10 (15.1%)	9 (13.6%)	5 (7.5%)	
Birth Interval (years)	First born	44 (66.6%)	46 (69.6%)	49 (74.2%)	-
	<1	13 (19.6%)	14 (21.2%)	9 (13.6%)	
	1–2	8 (12.1%)	3 (4.5%)	6 (9.0%)	
	2–3	1(1.5%)	2 (3.0%)	1 (1.5%)	
	>3	0	1 (1.5%)	1 (1.5%)	
	Don’t know	0	0	0	
Gravidity	<5	64 (96.9%)	61 (92.4%)	62 (93.9%)	1.34/0.50
	>5	2 (3.0%)	5 (7.5%)	4 (6.0%)	
Parity	<5	61 (92.4%)	64 (96.9%)	63 (95.4%)	1.28/0.47
	>5	3 (4.5%)	1 (1.5%)	1 (1.5%)	
	Don’t know	2 (3.0%)	1 (1.5%)	2 (3.0%)	
** Any other chronic disease	Yes	0	0	1(1.51%)	-
	No	66 (100%)	66 (100%)	65 (98.4%)	
Any special meal taken during pregnancy	Yes	64 (96.9%)	61 (92.4%)	59 (89.3%)	2.92/0.23
	No	2 (3.0%)	5 (7.5%)	7 (10.6%)	
Are you aware that your child is malnourished	Yes	61 (92.4%)	60 (90.9%)	49 (74.2%)	2.48/0.45
	No	5 (7.5%)	6 (9.0%)	17 (25.7%)	
Did you receive any antenatal care during this pregnancy	Yes	65 (98.4%)	65 (98.4%)	59 (89.3%)	8.38/0.15
	No	1 (1.5%)	1 (1.5%)	7 (10.6%)	
Were you given iron and folic acid tablets	Yes	66 (100%)	65 (98.4%)	62 (93.9%)	-
	No	0	1 (1.5%)	4 (6.0%)	
How many iron and folic acid tablets have you consumed	<30	62 (93.9%)	52 (78.7%)	48 (72.7%)	-
	30–60	4 (6.0%)	9 (13.6%)	7 (10.6%)	
	60–90	0	4 (6.0%)	6 (9.0%)	
	>90	0	1 (1.5%)	4 (6.0%)	
	Not consumed	0	0	(1.5%)	

** Chronic diseases include high blood pressure (1), gestational diabetes, preeclampsia, depression, and anxiety.

**Table 3:** Childbirth and illness histories of the study participants.

Variables	Characters	Control (*n*= 66)	Uncomplicated SAM (*n* = 66)	Complicated SAM (*n* = 66)	χ^2^	*p*-Value
Child age (months)	6–20	2 (3.0%)	10 (15.1)	49 (74.2%)	95.11	<0.001
	21–40	59 (89.3%)	51 (77.2%)	11 (16.6%)		
	41–60	5 (7.5%)	5 (7.5%)	6 (9.0%)		
Child birth history	Preterm	2 (3.0%)	4 (6.0%)	7 (10.6%)	2.65	0.58
	Full term	64 (96.9%)	62 (93.9%)	59 (89.3%)		
Birth weight (g)	<2000	6 (9.0%)	8 (12.1%)	13 (19.6%)	-	
	2000–25,000	58 (87.8%)	51 (77.2%)	51 (77.2%)		
	>25,000	2 (3.0%)	7 (10.6%)	1 (1.5%)		
	Don’t know	0	0	1 (1.5%)		
Child illness history within two weeks before assessment (*n* = 66 for each group)						
Diarrhea	Yes	0	7 (10.6%)	13(19.6%)	-	
	No	66 (100%)	59 (89.3%)	53 (80.3%)		
History of recurrent/chronic diarrhea	Yes	0	1 (1.5%)	5 (7.5%)	-	
	No	66 (100%)	65 (98.4%)	61 (92.4%)		
Fever	Yes	0	0	65(98.4%)	-	
	No	66 (100%)	66 (100%)	1(1.5%)		
Worm infection (Ascaris lumbricoids)	Yes	1 (1.5%)	2 (3.0%)	7 (10.6%)	1.28	0.54
	No	65 (98.4%)	64 (96.9%)	59 (89.3%)		
Cold and Cough	Yes	0	0	1 (1.5%)	-	
	No	66 (100%)	66 (100%)	65 (98.4%)		
Initiation of breastfeeding	After 1 h	65 (98.4%)	56 (84.8%)	51 (77.2%)	13.37	<0.001
	Within 1 h	1 (1.5%)	10 (15.1%)	15 (22.7%)		
Bottle feeding	Yes	56 (84.8%)	52 (78.7%)	24 (36.3%)	41.45	<0.001
	No	10 (15.1%)	14 (21.2%)	42 (63.6%)		
Pre-lactral feed	Yes	61 (92.4%)	48 (72.7%)	40 (60.6%)	14.67	<0.001
	No	5 (7.5%)	18 (27.2%)	26 (39.3%)		
Number of daily meals	<3	62 (93.9%)	61(92.4%)	59 (89.3%)	25.64	<0.001
	>3	4 (6.0%)	5 (7.5%)	7 (10.6%)		
Water consumption in a day (mL)	<500	62 (93.9%)	64(96.9%)	60 (90.9%)	12.78	<0.001
	>500	4 (6.0%)	2 (3.0%)	6 (9.0%)		
Urination (*n* = 66 for each group)						
Day	<3 times	17 (25.7%)	18 (27.2%)	29 (43.9%)	38.76	<0.001
	>3 times	49 (74.2%)	48 (72.7%)	37 (56.0%)		
Night	<3 times	24 (36.3%)	26 (39.3%)	47 (71.2%)	8.77	0.69
	>3 times	42 (63.6%)	40 (60.6%)	19 (28.7%)		
Time of eating initiation (months)	<12	66 (100%)	65 (98.4%)	65 (98.4%)	-	
	>12	0	1 (1.5%)	1(1.5%)		
Defecation (per day)	<2	62 (93.9%)	53 (80.3%)	56 (84.8%)	13.88	0.87
	>2	4 (6.0%)	13 (19.6%)	10 (15.1%)		
Family history of malnutrition	Yes	0	1 (1.5%)	2 (3.0%)	-	
	No	66 (100%)	65 (98.4%)	64(96.9%)		

**Table 4:** Biochemical parameters among the SAM children with and without complications and the control group.

Variables	Characters	Control (*n* = 66)	Uncomplicated SAM (*n* = 66)	Complicated SAM (*n* = 66)	ANOVA with *p*-Values *
		Mean ± SD	Mean ± SD	Mean ± SD	
Complete blood count	Hemoglobin (Hb) (g/dL)	10.69 ± 1.18	10.11 ± 1.74	7.75 ± 1.02	0.001 *
	Total Leukocyte Cell (TLC) (cells/mm^3^)	11,632.54 ± 2115.41	13,478.33 ± 3540.91	15,632.18 ± 2896.11	0.001 *
	Platelet count (lac cells/mm^3^)	3.89 ± 1.65	3.49 ± 1.15	3.36 ± 0.96	0.001 *
	Total RBCs (m cells/mm^3^)	4.39 ± 0.69	4.21 ± 0.42	4.07 ± 0.69	0.001 *
	Mean platelet volume (MPV)(fl.)	8.88 ± 2.01	8.91 ± 1.19	8.06 ± 0.96	0.001 *
Kidney function test	Serum Urea(mg/dL)	26.22 ± 1.89	28.01 ± 4.96	29.06 ± 2.11	0.001 *
	Serum Creatinine (mg/dL)	0.56 ± 0.18	0.81 ± 0.12	0.84 ± 0.19	0.001 *
Liver function test	Serum Bilrubin Total	0.36 ± 0.09	0.48 ± 0.12	0.55 ± 0.17	0.001 *
	Serum Bilrubin direct	0.18 ± 0.11	0.21 ± 0.07	0.32 ± 0.09	0.001 *
	Serum Bilirubin Indirect	0.19 ± 0.05	0.26 ± 0.08	0.29 ± 0.03	0.001 *
	Serum Protein	6.02 ± 0.08	7.1 ± 0.67	7.26 ± 0.05	0.001 *
	Serum Albumin	3.19 ± 0.14	4.52 ± 0.28	4.89 ± 0.33	0.001 *
	SGOT(IU/L)	39.56 ± 0.56	46.95 ± 0.52	47.88 ± 0.23	0.001 *
	SGPT(IU/L)	29.44 ± 14.26	32.16 ± 16.11	35.89 ± 14.32	0.001 *

* ANOVA with a *p*-Value > 0.05 was considered significant.

**Table 5:** Anthropometric outcome comparison for those who received vs did not receive antibiotics in children with uncomplicated SAM.

Study Variable	Baseline (*n* = 66)	SAM Children on the 15th Day of Antibiotic Interventions		
		Received Antibiotics (Mean ± SD) (*n* = 33)	Did not Receive Antibiotics (Mean ± SD) (*n* = 33)	*p*-Value
Weight (kg)	7.31 ± 1.6	7.9 ± 2.0	7.6 ± 1.7 7	<0.05
Height (cm)	74.73 ± 9.8	75.03 ± 9.7	74.73 ± 9.8	-
Weight-for-Height Z score	−2.8 ± 1.4	−1.93 ± 1.2	−2.29 ± 1.7	<0.05
Weight-for-age Z score	−4.4 ± 1.7	−3.50 ± 1.88	−4.0 ± 2.4	<0.05

## Data Availability

Data are contained within the article.
